# MST kinases in innate immune signaling

**DOI:** 10.15698/cst2018.01.119

**Published:** 2017-12-23

**Authors:** Zhubing Shi, Zhaocai Zhou

**Affiliations:** 1 State Key Laboratory of Cell Biology, CAS Center for Excellence in Molecular Cell Science, Institute of Biochemistry and Cell Biology, Shanghai Institutes for Biological Sciences, Chinese Academy of Sciences, Shanghai, China.; 2 School of Life Science and Technology, ShanghaiTech University, Shanghai, China.

**Keywords:** MST kinases, innate immunity, TLR signaling, antiviral signaling

## Abstract

The mammalian STE20-like (MST) protein kinases are composed of MST1, MST2, MST3, MST4 and YSK1. They play crucial roles in cell growth, migration, polarity and apoptosis. Dysfunction of these kinases often leads to diseases. MST kinases are extensively involved in development and function of immune system. Here, we review recent progresses on the regulatory function of MST kinases in innate immune signaling.

## INTRODUCTION

The MST kinases are evolutionarily conserved homologues of yeast Sterile20 (STE20) kinase 1. Mammalian STE20 kinases can be divided into GCK (germinal center kinase) and PAK (p21-activated kinase) families 2. The GCK family includes eight subfamilies, named GCKI to GCKVIII. They share a conserved kinase domain at N-terminal region, but possess diverse C-terminal regions that mediate protein-protein interaction. In mammal, the GCKII subfamily consists of MST1 (also named STK4) and MST2 (also named STK3); the GCKIII subfamily consists of MST3 (also named STK24), MST4 (also named STK26 or MASK) and YSK1 (also named STK25 and SOK1). The GCKII and GCKIII subfamilies constitute MST kinases.

MST1 and MST2 are major components of the mammalian Hippo signaling pathway, which controls cell number, organ size and tissue homeostasis 3,4,5,6,7,8. Together with SAV1 and MOB1A/B, MST1/2 can activate downstream kinases LATS1/2, leading to the phosphorylation and inhibition of the transcriptional coactivators YAP and TAZ. Unphosphorylated YAP/TAZ can freely translocate from the cytoplasm to the nucleus and then activate TEAD family transcription factors, causing the expression of pro-proliferative and anti-apoptotic genes. Therefore, MST1/2 are considered as tumor suppressors, and YAP/TAZ as oncoproteins. Upregulation of YAP or/and TAZ has been identified in many types of cancers, such as breast, colorectal, gastric, liver and lung cancers 9,10.

MST3, MST4 and YSK1 are implicated in the regulation of cell growth, migration, polarity and apoptosis. MST3 was firstly identified as a protein involved in caspase-mediated apoptosis 11. Following studies found that MST3 could phosphorylate NDR1/2, which are paralogs of LATS1/2 and also the substrates of MST1/2, to regulate cell cycle progression 12,13,14. MST3 promotes dendritic filopodia and spine synapse development via phosphorylating TAO1/2 15, while the MST3b isoform regulates axon regeneration 16. One of the major physiological functions of MST4 is acting downstream of the LKB1−MO25−STRAD polarization complex, which also works as tumor suppressor, to induce brush border formation in intestinal epithelial cells 17. YSK1 can function with LKB1 together, regulating the neuronal polarization via influencing the dynamics of the GM130-mediated Golgi apparatus 18,19. The LKB1−STRAD scaffold and activator MO25 can directly bind GCKIII kinases and significantly enhance their activities 20,21,22. However, the functional interplay between GCKIII kinases and LKB1 has not been fully addressed.

Recently, MST3 and MST4 were reported to promote cancer cell migration together with their adaptor CCM3 23,24,25,26. MST kinases, as well as CCM3, are components of supramolecular complexes termed "striatin (STRN)-interacting phosphatase and kinase (STRIPAK)" that contain both MST kinases and phosphatase PP2A with STRN proteins as scaffolds for complex assembly 27. STRN proteins recruit PP2A to negatively regulate the functions of MST kinases in cell growth and cancer metastasis most likely via dephosphorylating these kinases and therefore inhibition of their kinase activities 23,28.

Besides their roles in development and tumorigenesis, the MST kinases have been increasingly implicated in immune response, especially innate immune signaling during the last few years. These kinases modulate antimicrobial and antiviral responses at multi-levels. Here, we firstly introduce the known functions of MST kinases in immune system, and then summarize recent progresses on the regulatory relationship between MST kinases and innate immune signaling.

## FUNCTIONS OF MST KINASES IN IMMUNE SYSTEM

MST kinases especially MST1/2 are extensively involved in immune regulation 29. MST1 is enriched in lymphoid organs including thymus, spleen and lymph nodes 30. It regulates the proliferation of naïve T cells together with its partner RAPL (also named RASSF5 or NORE1). It is also suggested that MST1 protects naïve T cells from oxidative stress via phosphorylating and activating transcription factors FOXO1/3 31,32. Lymphocyte trafficking is a central event during immunological response and controlled by chemokines and adhesion receptors such as integrins 33. Upon sphingosine-1 phosphate and chemokine stimulation, MST1/2 governs Rho family GTPase Rac1 activation via phosphorylating MOB1A/B to promote thymocyte egress 34,35. The homing and egress abilities of T lymphocytes to peripheral lymphoid organs were impaired in *MST1* or *MST1/2* knockout mice 34,35,36. MST1 can be regulated by Rap1−RAPL signal to promote lymphocyte polarization and adhesion through inducing the translocation of LFA-1 (also named αLβ2 integrin) to the leading edge and immunological synapse 37,38,39. Recently, the well-known substrates NDR1/2 kinases and newly identified substrate L-plastin of MST1 were considered to mediate its function on thymocyte egress and T cell migration 40,41,42.

Treg cells are required for maintaining immune tolerance and homeostasis via inhibiting immune response of other cells 43. Treg cells can limit the development of autoimmune and chronic inflammatory diseases, and also have negative effects on cancer immunity. MST1/2 can regulate the development and function of Treg cells through phosphorylating and stabilizing FOXO1/3 44,45. FOXP3 is a specific marker of Treg cells and essential for their function 46. The function of FOXP3 is regulated by acetylation 47. Both lysine acetyltransferases TIP60 and p300 can promote FOXP3 acetylation and stability to mediate its function on transcriptional repression, while lysine deacetylases SIRT1 and HDAC6 have an opposite role. MST1 can enhance FOXP3 acetylation through suppressing the deacetylase activity of SIRT1 and the interaction between SIRT1 and FOXP3 48. Recent study found that the MST1/2 downstream effector TAZ suppresses FOXP3 acetylation and promotes its degradation, and therefore attenuates Treg cell differentiation 49. Th17 cells can protect the host against infection via its pro-inflammatory role. Geng *et al*. found that TAZ functions as a coactivator of the transcription factor RORγt, but not TEADs, to induce Th17 cell differentiation. However, TEAD1 can sequester TAZ from RORγt and FOXP3 to counterturn its function and promote Treg cell differentiation. Moreover, the MST1/2 kinases are supposed to be involved in these processes. Thus, both the MST1/2 kinases and the TAZ-TEAD transcription factors in the Hippo pathway play key roles in the maintenance of T cell homeostasis.

Neutrophils constitute the most part of white blood cells in circulation. Upon pathogen invasion, neutrophils migrate to inflammatory sites and execute the program of degranulation to release granular antimicrobial molecules 50. MST3 and its partner CCM3 can regulate neutrophil degranulation via modulating UNC13D-driven vesicle exocytosis 51. Furthermore, MST1-dependent vesicle trafficking is required for neutrophil extravasation 52.

## INNATE IMMUNE SIGNALING

Innate immunity is the first line of defense against pathogen invasion. Innate immune system utilizes multiple pathogen pattern receptors to sense pathogen-associated molecular patterns (PAMPs) from pathogens and trigger immune response. Pathogen pattern receptors include membrane-anchored Toll-like receptors (TLRs) and C-type lectin receptors, and cytosolic RIG-I-like receptors (RLRs), NOD-like receptors and AIM2-like receptors 53,54. TLR- and RLR-mediated signaling has been reported to associate with MST kinases. There are ten TLRs in humans. TLR1, TLR2, TLR4, TLR5, TLR6 and TLR10 are localized on plasma membrane where they sense mainly microbial membrane components, while TLR3, TLR7, TLR8 and TLR 9 are expressed in endosomal compartments where they recognize microbial nucleic acids 55,56. TLR2 can sense multiple microbial components such as lipoproteins, peptidoglycans and hemagglutinin from bacteria, fungi and viruses. TLR2 usually functions via forming heterodimers with other TLRs such as TLR1 and TLR6. TLR4 recognizes bacterial lipopolysaccharide (LPS). TLR5 is a receptor for bacterial flagellin. Endosomal TLR3 recognizes viral double-stranded RNA (dsRNA) and its synthetic analogue polyinosinic-polycytidylic acid (poly(I:C)), while TLR7 and TLR8 recognize viral single-stranded RNA. TLR9 specifically senses unmethylated CpG motifs in bacterial and viral DNA.

**Figure 1:**
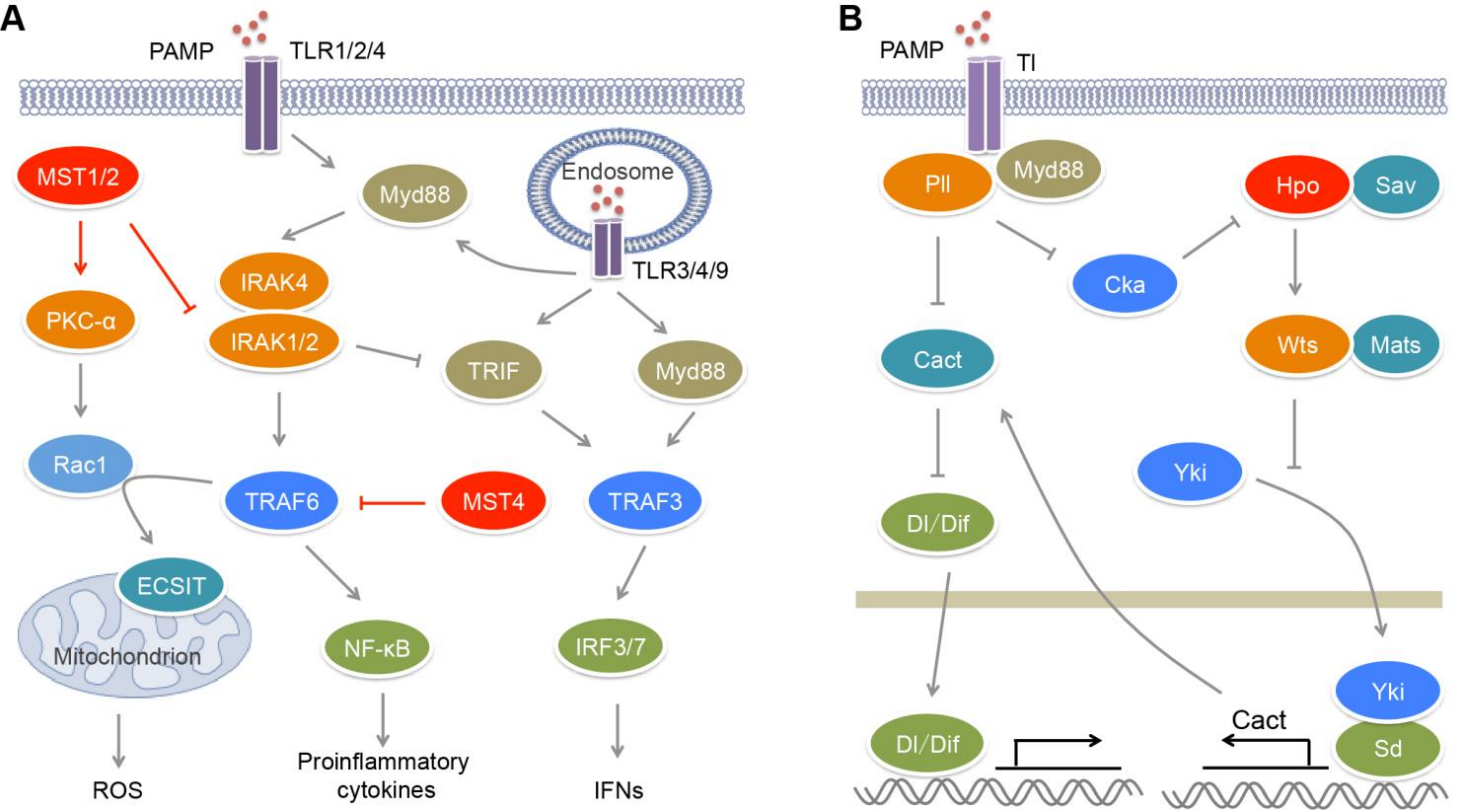
FIGURE 1: MST kinases in the regulation of TLR signaling. **(A)** In mammals, upon microbial infection, TLRs sense different kinds of PAMPs and promote the production of pro-inflammatory cytokines and IFNs through activating E3 ligases TRAF3/6 and transcription factors NF-κB and IRF3/7. TRAF6 also promotes mROS production in a Rac1- and ECSIT-dependent manner. MST1/2 could stimulate mROS production through PKC-α−Rac1−TRAF6−ECSIT pathway to clear pathogens. On the contrary, MST1 suppresses the upstream kinase IRAK1 of TRAF6 to limit the production of pro-inflammatory cytokines. MST4 also attenuates TLR-mediated inflammatory responses through directly phosphorylating and inhibiting TRAF6. The inhibitory roles of MST1 and MST4 in proinflammatory response protect body against inflammatory damage and chronic inflammation-driven HCC. **(B)** In *Drosophila*, upon microbial infection, Tl receptor promotes the activation of Hpo kinase, which in turn restricts the transcription of Dl/Dif inhibitor Cact through Hippo signaling, and thus promotes Tl-mediated antimicrobial response.

Upon activated by their ligands, TLRs can recruit adaptors MyD88 or/and TRIF that bind different downstream signal molecules 55 (**Figure 1A**). MyD88 recruits IRAK4, IRAK1 and IRAK2 forming the Myddosome, which bind and activate E3 ligase TRAF6. TRAF6 in turn activates the IKK complex including NEMO, IKKα and IKKβ, which phosphorylates IκB and promotes its degradation, causing the activation of transcription factor NF-κB and the induction of pro-inflammatory cytokines. TRIF can recruit E3 ligase TRAF3, resulting in the activation of transcription factor IRF3 and the induction of type I interferons (IFNs). The TLR2−TLR1/TLR6 heterodimers, TLR5, TLR7, TLR8 and TLR9 recruit MyD88 to activate NF-κB, while TLR3 recruits TRIF to activate IRF3. TLR7, TLR8 and TLR9 also induce MyD88-mediated TRAF3 activation that causes IRF7-mediated transcription. The activation of TLR4 induces Myddosome formation and NF-κB activation. In the help of CD14, TLR4 can translocate to endosome where it recruits TRIF and activates both IRF3 and NF-κB.

**Figure 2:**
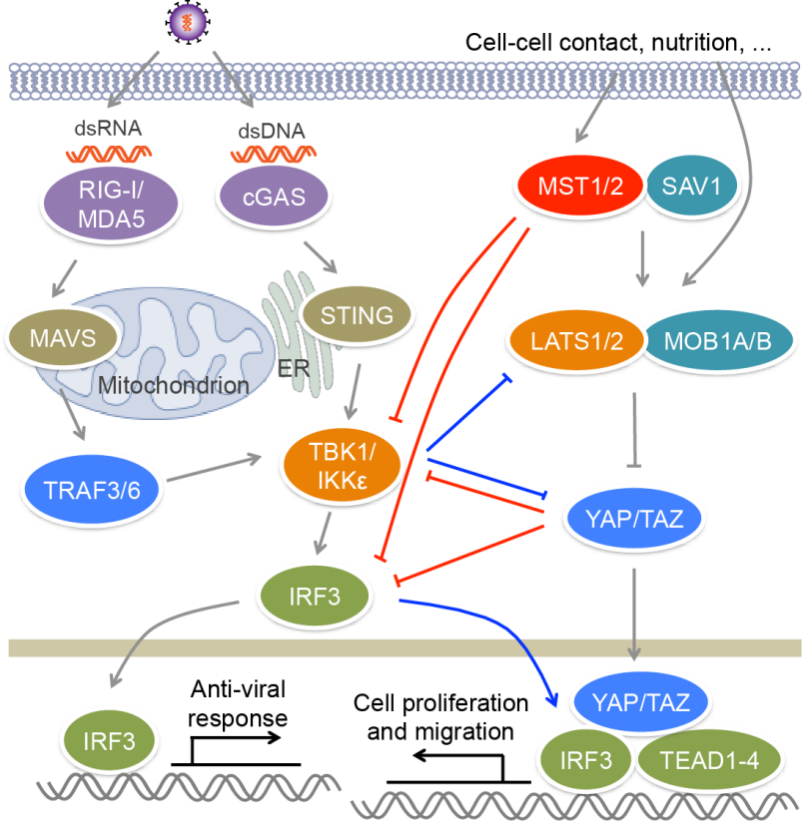
FIGURE 2: MST kinases and Hippo signaling regulate antiviral signaling. Upon viral infection, RIG-I/MDA5 and cGAS recognize viral dsRNA and dsDNA respectively, and activate TBK1/IKKε via adaptors MAVS and TRIF, as well as E3 ligases TRAF3/6, leading to the activation of IRF3 and the production of IFNs. MST1 can phosphorylate and suppress the activation of IRF3, as well as TBK1, to impair antiviral response. The downstream effectors YAP/TAZ of the Hippo pathway also negatively regulate antiviral signaling through inactivating TBK1 and IRF3. Viral infection induces the phosphorylation and degradation of YAP by IKKε, relieving its inhibitory role in antiviral immunity. IKKε also induce the ubiquitination and degradation of LATS1/2 in glioblastoma multiforme cell lines, causing YAP activation. In addition, IRF3 can promote YAP−TEAD-driven gastric caner growth.

RLRs are composed of three members, RIG-I, MDA5 and LGP2. RIG-I and MDA5 recognize viral dsRNA 57 (**Figure 2**). RIG-I prefers short 5’ppp and 5’pp dsRNA, while MDA5 recognizes long dsRNA. During viral infection, activated RIG-I and MDA5 bind to a mitochondrion-located adaptor MAVS and induce its oligomerization 58. The resultant MAVS filament further recruits multiple TRAF proteins such as TRAF2, TRAF3 and TRAF6 to activate TBK1, IKKε, IKKα and IKKβ, leading to the activation of IRF3 and NF-κB and the production of type I and type III IFNs. Cytosolic DNA sensors include cGAS, IFI16, DAI and DDX41 59. Viral DNA binds and activates cGAS, which utilizes ATP and GTP to generate cyclic di-GMP/AMP 60 (**Figure 2**). Cyclic di-GMP/AMP induces endoplasmic reticulum-resident STING dimerization to activate TBK1−IRF3 signaling, causing the production of antiviral type I IFNs.

## MST KINASES AND TLR SIGNALING

Recently, we and others have reported that MST kinases play crucial roles on the modulation of TLR signaling and inflammation 61,62,63 (**Figure 1A**). The expression of MST4 responds dynamically to LPS stimulation and/or bacterial infection in immune cells and organs of mice 61. Overexpression of MST4 suppresses LPS- or bacteria-induced production of pro-inflammatory cytokines IL-6 and TNF-α in human THP-1 cells and mouse peritoneal exudate macrophages (PEMs), while knockdown of MST4 has an opposite role. MST4 activator MO25 can further enhance the inhibitory effect of MST4 on TLR signaling. Upon LPS stimulation, MST4 expression is increased, and it directly binds and phosphorylates the TRAF_C domain of TRAF6 at Thr463 and Thr486, which impairs the oligomerization of TRAF6. Due to the important role of oligomerization on the E3 ligase activity of TRAF6 64,65, TRAF6 phosphorylation by MST4 impairs its autoubiquitination and the formation of signaling complex, causing the suppression of TLR-mediated inflammatory responses. Clinical data showed that MST4 expression is markedly downregulated and inversely correlates with IL6 expression in the peripheral blood samples of patients with sepsis 61. In a mouse model developing septic shock, MST4 protects mice against exacerbated inflammation in a TRAF6- and macrophage-dependent manner, suggesting that MST4 has a crucial role in limiting inflammatory damage and maintaining immune homeostasis upon bacterial infection.

During pathogen infection, phagocytes such as neutrophils and macrophages can recognize, engulf, and degrade microorganism 66,67,68. Phagocytes sense chemical components and physical properties of microorganism and engulf microorganism into phagosomes. Phagosomal NADPH oxidase machinery can generate reactive oxygen species (ROS) to kill pathogens. Mitochondrial ROS (mROS) is required for the optimal clearance of pathogen by macrophages 69 (**Figure 1A**). Cell surface receptors TLR1/2/4 contributes to the mROS production via recruiting mitochondria to phagosomes. A further study found that MST1/2 are indispensible for this process (**Figure 1A**). Depletion of MST1/2 in myeloid cells leads to more susceptibility of mice to bacterial infection and severe inflammation 62. Upon bacterial infection, MST1/2 are activated by TLR1/2/4−MyD88 signaling and in turn phosphorylates PKC-α at Ser226 and Thr228, leading to PKC-α activation. Activated PKC-α interferes with the binding of the Rho-GTP-dissociation inhibitor Ly-GDI (also named Rho-GDI2) to Rac1 via phosphorylating Ly-GDI at Ser31, leading to the release of Rac1 70. TRAF6 can further mediate Lys63-linked polyubiquitination of GTP-charged Rac1 at Lys16 that results in its activation. In turn, the polyubiquitination of Rac1 promotes the interaction of TRAF6 with ECSIT. TRAF6 enhances ECSIT ubiquitination and enrichment at the mitochondrial periphery 69. ECSIT is a regulator of mitochondrial complex I assembly 71. It associates with mitochondrial complex I assembly chaperone NDUFAF1 to regulate mitochondrial function that is required for the recruitment of mitochondria to phagosomes and the production of ROS in phagocytes. However, the mechanism for the activation of MST1/2 by TLRs is still undefined. Recently, Liu *et al*. found that *Drosophila* IRAK homolog Pelle (Pll) acting downstream of Toll (Tl) receptor directly phosphorylates Cka (*Drosophila* homolog of STRNs) and promotes its degradation 72 (**Figure 1B**). Because both the kinase Hippo (Hpo, *Drosophila* homolog of MST1/2) and the phosphatase PP2A B’’’ regulatory subunit Cka are major components of *Drosophila* STRIPAK complex and Cka is a negative regulator of Hpo activity 28, the degradation of Cka will facilitate the activation of Hpo. Thus mammalian MST1/2 may be activated by TLR1/2/4 in a similar manner.

Besides their roles on TLR-mediated ROS production, MST1/2 are also involved in the regulation of canonical TLR-mediated inflammatory response (**Figure 1A**). Upon the stimulation with poly(I:C), LPS and CpG that activate TLR3/4/9 respectively, the MST1 expression is decreased in mouse PEMs 63. MST1 deficiency results in increased expression of IL-1β, IL-6 and TNF-α in macrophages in response to LPS and CpG stimulation, but reduced expression of IFN-β in response to LPS and poly(I:C) stimulation 62,63. IRAK1 is a component of the Myddsome and required for TLR-mediated production of pro-inflammatory cytokines 73. However, its phosphorylation and degradation promotes IFN-β production 74,75. MST1 can associate with IRAK1 to induce its phosphorylation and degradation, resulting in impaired TLR4/9-mediated NF-κB activation and pro-inflammatory cytokines production but enhanced TLR3/4-stimulated IRF3 phosphorylation and IFN-β production 63. Whether IRAK1 is the direct substrate of MST1 needs more biochemical investigation.

Numerous studies have revealed the regulatory roles of TLR signaling on cancer development 76,77. Many TLRs ligands such as poly(I:C), LPS and CpG have potential antitumor activity, indicating the activation of TLRs suppresses tumorigenesis might through stimulating IFN response. However, other reports suggest TLR-mediated chronic inflammation promotes tumor development. For example, ultraviolet irradiation promotes angiotropism and metastasis in melanoma through inducing TLR4/Myd88-driven neutrophilic inflammation 78. Intestinal microbiota and TLR4 signaling also have potential roles in promoting hepatocellular carcinoma (HCC) proliferation 79. Li *et al*. found that the expression of MST1 is significantly decreased in macrophages from HCC patients and inversely correlated with the expression of IRAK1, and the levels of phosphorylated p65 and STAT3 63. Consistent with its inhibitory role in pro-inflammatory response and positive role in IFN response, MST1 guards mice from chronic inflammation-driven HCC upon LPS stimulation or bacterial infection.

Besides the direct regulation of TLR signaling, the GCKII family kinases also facilitates TLR-mediated antimicrobial response via Hippo signaling (**Figure 1B**). In *Drosophila*, like Tl and Myd88 deficiency, depletion of Hpo or Warts (Wts, *Drosophila* homolog of LATS1/2) in fly fat bodies, as well as overexpression of Yorkie (Yki, *Drosophila* homolog of YAP), causes higher sensitivity to bacterial and fungal infection 72. The transcription of *Drosophila* IκB homolog Cactus (Cact) that inhibits the NF-κB family transcription factors Dorsal (Dl) and Dorsal-related immune factor (Dif) is controlled by the Yki-Scalloped (Sd, *Drosophila* homolog of TEADs) transcription factor complex. Hpo can suppress the expression of Cact to promote innate immune response via inhibiting Yki activity. Due to the impact of Tl on Hpo activation, microbial infection can trigger Hippo signal transduction, which inhibits Cact expression and subsequently facilitates antimicrobial response.

## MST KINASES IN ANTIVIRAL SIGNALING REGULATION

The activation of transcription factor IRF3 is a key step in antiviral response. IRF3 is constituted of an N-terminal DNA binding domain, an IRF association domain and a C-terminal auto-inhibitory region. Upon viral infection, IRF3 is phosphorylated by TBK1 or IKKε multiple sites in auto-inhibitory region, which leads to its homo-dimerization and nuclear localization 80,81. In nucleus, IRF3 binds the IFN-stimulated response element (ISRE) sequences within the promoters of IFN genes to activate their transcription. MST1 was reported to suppress RIG-I−MAVS- and cGAS−STING-mediated antiviral response via the regulation of IRF3, as well as its upstream kinase TBK1 82 (**Figure 2**). MST1 can interact with and phosphorylate IRF3 at Thr75 and Thr253. MST1 partners SAV1 and RASSF family proteins RASSF1A and RASSF5 may mediate the interaction between MST1 and IRF3 to promote IRF3 phosphorylation. Residues Thr75 and Thr253 localize at DNA binding and IRF association domains of IRF3 respectively. Phosphorylation of these two threonine residues disrupts the dimerization and DNA-binding ability of IRF3 and thus impairs its transcription activity and antiviral signaling. Furthermore, PPM1B was identified as the phosphatase responsible for dephosphorylation of IRF3 at Thr75 and Thr253 82. MST1 also suppresses TBK1 activation, further contributing to the attenuation of IRF3 activation. Zebrafish ectopically expressing MST1 are more susceptible to viral infection, while depletion of MST1 protects cells and mice against viral infection, suggesting MST1 physiologically suppresses innate immune response during viral invasion, which may prevent tissue damage caused by excessive IFN response.

Recently, two groups reported that YAP/TAZ in the Hippo pathway could negatively regulate antiviral response 83,84 (**Figure 2**). Zhang *et al*. found that YAP/TAZ attenuate K63-linked polyuniquitination of TBK1 by TRAFs, and they also associate with TBK1/IKKε to suppress their binding to upstream adaptors STING and MAVS, as well as to the substrate IRF3, both of which result in the inhibition of TBK1 activity and IRF3 phosphorylation 83. Distinct from this finding, Wang *et al*. reported that YAP associates with IRF3 to directly block its homo-dimerization and interaction with importins, and therefore to prevent its nuclear translocation, but has no influence on the activity of TBK1/IKKε the phosphorylation of IRF3 84. Both of groups demonstrated that the transcriptional activity of YAP/TAZ is dispensable for their inhibitory role in antiviral response, but the C-terminal transactivation of YAP is required for this function. Overexpressing YAP or TAZ enhances viral infection in human cells and zebrafish, while myeloid YAP deficiency has an opposite effect in mice. When Hippo signal is turned "ON" by cellular nutrition or physical status including serum starvation and high cell confluence, YAP/TAZ are phosphorylated and inactivated by MST1/2 downstream kinases LATS1/2, and thus their suppression on TBK1 is relieved 83. This finding is different from the aforementioned role of MST1 in antiviral response through directly inhibiting IRF3 that does not depend on Hippo signaling 82. Furthermore, viral infection induces the phosphorylation of YAP at Ser403 by IKKε, triggering the lysosomal degradation of YAP and also relieving its negative function in antiviral immunity, which is independent of Hippo signaling 84. However, IKKε was also reported to induce the ubiquitination and proteasomal degradation of LATS1/2 in glioblastoma multiforme cell lines, causing YAP activation 85. A selective inhibitor of IKKε, amlexanox, reverses the inhibition of IKKε on LATS1/2 and thus declines glioblastoma cell migration and invasion and glioblastoma growth in xenograft mouse model. In this regard, we have recently found that both IRF3 and YAP are unregulated in clinical samples of gastric cancer 86. Viral infection that activates IRF3 relieves the inhibition of YAP. Mechanistically, phosphorylated IRF3 can bind both YAP and TEAD4 in the nucleus to co-regulate the target genes of the Hippo pathway. Treatment with amlexanox that decreases the activity of IRF3, significantly suppresses YAP-driven gastric cancer growth in mice. These observations further indicate a complex interplay between the Hippo pathway and innate immune regulation.

## CONCLUSION AND PERSPECTIVE

MST kinases are emerging as crucial regulators of innate immune response. They regulate antibacterial and antiviral signaling via targeting multiple molecules including kinases IRAK1 and TBK1/IKKε, E3 ligase TRAF6 and transcription factor IRF3. The activity of these signal molecules is important for their functions and must be strictly regulated. Current results suggest that MST kinases can suppress the activity of these molecules via direct phosphorylation, which is required for limiting excessive immune response. MST kinases also have positive roles against pathogen infection through diverse mechanism. Therefore, MST kinases and the Hippo pathway exert their influences on the regulation of innate immune response likely in a context-dependent manner. The type of pathogens and host cells, as well as the specific tissue microenvironment, might determine the positive or negative role of MST kinases in innate immunity. Given that the activation of MST kinases is regulated by cell−cell contact, mechanical cues, GPCR signal and cell stress 87,88, these factors may also influence innate immune response. Furthermore, the activity of MST kinases is negatively regulated by the PP2A module in the STRIPAK complex, so other components of STRIPAK complex are expected to regulate microbial defense programs. Several components of STRIPAK complex have been reported to participate in the regulation of T and B cell development and IFN signaling 89,90,91. Whether other subunits possess MST-dependent or independent roles on immune regulation need to be investigated.

Excessive inflammatory response usually causes tissue damage, while impaired immune response is insufficient to prevent microbial infection. Due to the important function on immune regulation, dysregulation of MST kinases often links to immune diseases. Several mutations of MST1 have been detected in patients with immunodeficiency 92,93. These patients are susceptive to bacterial and viral infection possibly caused by defective development of T and B cells. This observation is distinct from that in mice where MST1 deficiency resulted in impaired innate antiviral response 82. Thus MST1 might play distinct roles at different stages of antiviral immunity. In patients with IgG4-related autoimmune pancreatitis and rheumatoid arthritis, the CpG sites in the promoter region of MST1 were hypermethylated and the expression of MST1 was reduced in patient Treg cells 94. It is possible that the disorders caused by these alterations on MST1 partially result from the dysregulation of innate immune response. The association of MST1 and other MST kinases and immune diseases remains to be elucidated. Until now, studies on the regulatory roles of MST kinases in immune regulation are still limited. Future discoveries will uncover their novel regulatory mechanism and provide new therapeutic strategies for related diseases.

## Abbreviations

dsRNA: double-stranded RNA
GCK: germinal center kinase
HCC: hepatocellular carcinoma
IFN: interferon
LPS: lipopolysaccharide
mROS: mitochondrial ROS
Poly(I:C): polyinosinic-polycytidylic acid
RLR: RIG-I-like receptor
ROS: reactive oxygen species
STRN: striatin
TLR: Toll-like receptor

## References

[1] Manning G, Whyte DB, Martinez R, Hunter T, Sudarsanam S (2002). The protein kinase complement of the human genome.. Science.

[2] Strange K, Denton J, Nehrke K (2006). Ste20-type kinases: evolutionarily conserved regulators of ion transport and cell volume.. Physiology (Bethesda).

[3] Yu FX, Zhao B, Guan KL (2015). Hippo Pathway in Organ Size Control, Tissue Homeostasis, and Cancer.. Cell.

[4] Shi Z, Jiao S, Zhou Z (2015). Structural dissection of Hippo signaling.. Acta Biochim Biophys Sin (Shanghai).

[5] Zhou D, Conrad C, Xia F, Park JS, Payer B, Yin Y, Lauwers GY, Thasler W, Lee JT, Avruch J, Bardeesy N (2009). Mst1 and Mst2 maintain hepatocyte quiescence and suppress hepatocellular carcinoma development through inactivation of the Yap1 oncogene.. Cancer Cell.

[6] Song H, Mak KK, Topol L, Yun K, Hu J, Garrett L, Chen Y, Park O, Chang J, Simpson RM, Wang CY, Gao B, Jiang J, Yang Y (2010). Mammalian Mst1 and Mst2 kinases play essential roles in organ size control and tumor suppression.. Proc Natl Acad Sci U S A.

[7] Lu L, Li Y, Kim SM, Bossuyt W, Liu P, Qiu Q, Wang Y, Halder G, Finegold MJ, Lee JS, Johnson RL (2010). Hippo signaling is a potent in vivo growth and tumor suppressor pathway in the mammalian liver.. Proc Natl Acad Sci U S A.

[8] Zhou D, Zhang Y, Wu H, Barry E, Yin Y, Lawrence E, Dawson D, Willis JE, Markowitz SD, Camargo FD, Avruch J (2011). Mst1 and Mst2 protein kinases restrain intestinal stem cell proliferation and colonic tumorigenesis by inhibition of Yes-associated protein (Yap) overabundance.. Proc Natl Acad Sci U S A.

[9] Moroishi T, Hansen CG, Guan KL (2015). The emerging roles of YAP and TAZ in cancer.. Nat Rev Cancer.

[10] Zanconato F, Cordenonsi M, Piccolo S (2016). YAP/TAZ at the Roots of Cancer.. Cancer Cell.

[11] Huang CY, Wu YM, Hsu CY, Lee WS, Lai MD, Lu TJ, Huang CL, Leu TH, Shih HM, Fang HI, Robinson DR, Kung HJ, Yuan CJ (2002). Caspase activation of mammalian sterile 20-like kinase 3 (Mst3).. Nuclear translocation and induction of apoptosis. J Biol Chem.

[12] Cornils H, Kohler RS, Hergovich A, Hemmings BA (2011). Human NDR kinases control G(1)/S cell cycle transition by directly regulating p21 stability.. Mol Cell Biol.

[13] Stegert MR, Hergovich A, Tamaskovic R, Bichsel SJ, Hemmings BA (2005). Regulation of NDR protein kinase by hydrophobic motif phosphorylation mediated by the mammalian Ste20-like kinase MST3.. Mol Cell Biol.

[14] Vichalkovski A, Gresko E, Cornils H, Hergovich A, Schmitz D, Hemmings BA (2008). NDR kinase is activated by RASSF1A/MST1 in response to Fas receptor stimulation and promotes apoptosis.. Curr Biol.

[15] Ultanir SK, Yadav S, Hertz NT, Oses-Prieto JA, Claxton S, Burlingame AL, Shokat KM, Jan LY, Jan YN (2014). MST3 kinase phosphorylates TAO1/2 to enable Myosin Va function in promoting spine synapse development.. Neuron.

[16] Lorber B, Howe ML, Benowitz LI, Irwin N (2009). Mst3b, an Ste20-like kinase, regulates axon regeneration in mature CNS and PNS pathways.. Nat Neurosci.

[17] ten Klooster JP, Jansen M, Yuan J, Oorschot V, Begthel H, Di Giacomo V, Colland F, de Koning J, Maurice MM, Hornbeck P, Clevers H (2009). Mst4 and Ezrin induce brush borders downstream of the Lkb1/Strad/Mo25 polarization complex.. Dev Cell.

[18] Matsuki T, Matthews RT, Cooper JA, van der Brug MP, Cookson MR, Hardy JA, Olson EC, Howell BW (2010). Reelin and stk25 have opposing roles in neuronal polarization and dendritic Golgi deployment.. Cell.

[19] Preisinger C, Short B, De Corte V, Bruyneel E, Haas A, Kopajtich R, Gettemans J, Barr FA (2004). YSK1 is activated by the Golgi matrix protein GM130 and plays a role in cell migration through its substrate 14-3-3zeta.. J Cell Biol.

[20] Shi Z, Jiao S, Zhang Z, Ma M, Zhang Z, Chen C, Wang K, Wang H, Wang W, Zhang L, Zhao Y, Zhou Z (2013). Structure of the MST4 in complex with MO25 provides insights into its activation mechanism.. Structure.

[21] Filippi BM, de los Heros P, Mehellou Y, Navratilova I, Gourlay R, Deak M, Plater L, Toth R, Zeqiraj E, Alessi DR (2011). MO25 is a master regulator of SPAK/OSR1 and MST3/MST4/YSK1 protein kinases.. EMBO J.

[22] Hao Q, Feng M, Shi Z, Li C, Chen M, Wang W, Zhang M, Jiao S, Zhou Z (2014). Structural insights into regulatory mechanisms of MO25-mediated kinase activation.. J Struct Biol.

[23] Madsen CD, Hooper S, Tozluoglu M, Bruckbauer A, Fletcher G, Erler JT, Bates PA, Thompson B, Sahai E (2015). STRIPAK components determine mode of cancer cell migration and metastasis.. Nat Cell Biol.

[24] Ma X, Zhao H, Shan J, Long F, Chen Y, Chen Y, Zhang Y, Han X, Ma D (2007). PDCD10 interacts with Ste20-related kinase MST4 to promote cell growth and transformation via modulation of the ERK pathway.. Mol Biol Cell.

[25] Zhang M, Dong L, Shi Z, Jiao S, Zhang Z, Zhang W, Liu G, Chen C, Feng M, Hao Q, Wang W, Yin M, Zhao Y, Zhang L, Zhou Z (2013). Structural mechanism of CCM3 heterodimerization with GCKIII kinases.. Structure.

[26] Fidalgo M, Fraile M, Pires A, Force T, Pombo C, Zalvide J (2010). CCM3/PDCD10 stabilizes GCKIII proteins to promote Golgi assembly and cell orientation.. J Cell Sci.

[27] Shi Z, Jiao S, Zhou Z (2016). STRIPAK complexes in cell signaling and cancer.. Oncogene.

[28] Ribeiro PS, Josue F, Wepf A, Wehr MC, Rinner O, Kelly G, Tapon N, Gstaiger M (2010). Combined functional genomic and proteomic approaches identify a PP2A complex as a negative regulator of Hippo signaling.. Mol Cell.

[29] Du X, Yu A, Tao W (2015). The non-canonical Hippo/Mst pathway in lymphocyte development and functions.. Acta Biochim Biophys Sin (Shanghai).

[30] Zhou D, Medoff BD, Chen L, Li L, Zhang XF, Praskova M, Liu M, Landry A, Blumberg RS, Boussiotis VA, Xavier R, Avruch J (2008). The Nore1B/Mst1 complex restrains antigen receptor-induced proliferation of naive T cells.. Proc Natl Acad Sci U S A.

[31] Choi J, Oh S, Lee D, Oh HJ, Park JY, Lee SB, Lim DS (2009). Mst1-FoxO signaling protects Naive T lymphocytes from cellular oxidative stress in mice.. PLoS One.

[32] Lehtinen MK, Yuan Z, Boag PR, Yang Y, Villen J, Becker EB, DiBacco S, de la Iglesia N, Gygi S, Blackwell TK, Bonni A (2006). A conserved MST-FOXO signaling pathway mediates oxidative-stress responses and extends life span.. Cell.

[33] Kinashi T (2007). Integrin regulation of lymphocyte trafficking: lessons from structural and signaling studies.. Adv Immunol.

[34] Mou F, Praskova M, Xia F, Van Buren D, Hock H, Avruch J, Zhou D (2012). The Mst1 and Mst2 kinases control activation of rho family GTPases and thymic egress of mature thymocytes.. J Exp Med.

[35] Dong Y, Du X, Ye J, Han M, Xu T, Zhuang Y, Tao W (2009). A cell-intrinsic role for Mst1 in regulating thymocyte egress.. J Immunol.

[36] Katagiri K, Katakai T, Ebisuno Y, Ueda Y, Okada T, Kinashi T (2009). Mst1 controls lymphocyte trafficking and interstitial motility within lymph nodes.. EMBO J.

[37] Ueda Y, Katagiri K, Tomiyama T, Yasuda K, Habiro K, Katakai T, Ikehara S, Matsumoto M, Kinashi T (2012). Mst1 regulates integrin-dependent thymocyte trafficking and antigen recognition in the thymus.. Nat Commun.

[38] Katagiri K, Imamura M, Kinashi T (2006). Spatiotemporal regulation of the kinase Mst1 by binding protein RAPL is critical for lymphocyte polarity and adhesion.. Nat Immunol.

[39] Katagiri K, Maeda A, Shimonaka M, Kinashi T (2003). RAPL, a Rap1-binding molecule that mediates Rap1-induced adhesion through spatial regulation of LFA-1.. Nat Immunol.

[40] Tang F, Gill J, Ficht X, Barthlott T, Cornils H, Schmitz-Rohmer D, Hynx D, Zhou D, Zhang L, Xue G, Grzmil M, Yang Z, Hergovich A, Hollaender GA, Stein JV, Hemmings BA, Matthias P (2015). The kinases NDR1/2 act downstream of the Hippo homolog MST1 to mediate both egress of thymocytes from the thymus and lymphocyte motility.. Sci Signal.

[41] Xu X, Wang X, Todd EM, Jaeger ER, Vella JL, Mooren OL, Feng Y, Hu J, Cooper JA, Morley SC, Huang YH (2016). Mst1 Kinase Regulates the Actin-Bundling Protein L-Plastin To Promote T Cell Migration.. J Immunol.

[42] Kondo N, Ueda Y, Kita T, Ozawa M, Tomiyama T, Yasuda K, Lim DS, Kinashi T (2017). NDR1-Dependent Regulation of Kindlin-3 Controls High-Affinity LFA-1 Binding and Immune Synapse Organization.. Mol Cell Biol.

[43] Josefowicz SZ, Lu LF, Rudensky AY (2012). Regulatory T cells: mechanisms of differentiation and function.. Annu Rev Immunol.

[44] Tomiyama T, Ueda Y, Katakai T, Kondo N, Okazaki K, Kinashi T (2013). Antigen-specific suppression and immunological synapse formation by regulatory T cells require the Mst1 kinase.. PLoS One.

[45] Du X, Shi H, Li J, Dong Y, Liang J, Ye J, Kong S, Zhang S, Zhong T, Yuan Z, Xu T, Zhuang Y, Zheng B, Geng JG, Tao W (2014). Mst1/Mst2 regulate development and function of regulatory T cells through modulation of Foxo1/Foxo3 stability in autoimmune disease.. J Immunol.

[46] Ramsdell F, Ziegler SF (2014). FOXP3 and scurfy: how it all began.. Nat Rev Immunol.

[47] van Loosdregt J, Coffer PJ (2014). Post-translational modification networks regulating FOXP3 function.. Trends Immunol.

[48] Li J, Du X, Shi H, Deng K, Chi H, Tao W (2015). Mammalian Sterile 20-like Kinase 1 (Mst1) Enhances the Stability of Forkhead Box P3 (Foxp3) and the Function of Regulatory T Cells by Modulating Foxp3 Acetylation.. J Biol Chem.

[49] Geng J, Yu S, Zhao H, Sun X, Li X, Wang P, Xiong X, Hong L, Xie C, Gao J, Shi Y, Peng J, Johnson RL, Xiao N, Lu L, Han J, Zhou D, Chen L (2017). The transcriptional coactivator TAZ regulates reciprocal differentiation of TH17 cells and Treg cells.. Nat Immunol.

[50] Amulic B, Cazalet C, Hayes GL, Metzler KD, Zychlinsky A (2012). Neutrophil function: from mechanisms to disease.. Annu Rev Immunol.

[51] Zhang Y, Tang W, Zhang H, Niu X, Xu Y, Zhang J, Gao K, Pan W, Boggon TJ, Toomre D, Min W, Wu D (2013). A network of interactions enables CCM3 and STK24 to coordinate UNC13D-driven vesicle exocytosis in neutrophils.. Dev Cell.

[52] Kurz AR, Pruenster M, Rohwedder I, Ramadass M, Schafer K, Harrison U, Gouveia G, Nussbaum C, Immler R, Wiessner JR, Margraf A, Lim DS, Walzog B, Dietzel S, Moser M, Klein C, Vestweber D, Haas R, Catz SD, Sperandio M (2016). MST1-dependent vesicle trafficking regulates neutrophil transmigration through the vascular basement membrane.. J Clin Invest.

[53] Brubaker SW, Bonham KS, Zanoni I, Kagan JC (2015). Innate immune pattern recognition: a cell biological perspective.. Annu Rev Immunol.

[54] Akira S, Uematsu S, Takeuchi O (2006). Pathogen recognition and innate immunity.. Cell.

[55] Pandey S, Kawai T, Akira S (2015). Microbial sensing by Toll-like receptors and intracellular nucleic acid sensors.. Cold Spring Harb Perspect Biol.

[56] Kawai T, Akira S (2010). The role of pattern-recognition receptors in innate immunity: update on Toll-like receptors.. Nat Immunol.

[57] Loo YM, Gale Jr M (2011). Immune signaling by RIG-I-like receptors.. Immunity.

[58] Wu B, Hur S (2015). How RIG-I like receptors activate MAVS.. Curr Opin Virol.

[59] Goubau D, Deddouche S, Reis e Sousa C (2013). Cytosolic sensing of viruses.. Immunity.

[60] Ma Z, Damania B (2016). The cGAS-STING Defense Pathway and Its Counteraction by Viruses.. Cell Host Microbe.

[61] Jiao S, Zhang Z, Li C, Huang M, Shi ZW, Wang Y, Song X, Liu H, Li C, Chen M, Wang W, Zhao Y, Jiang Z, Wang H, Wong CC, Wang C, Zhou Z (2015). The kinase MST4 limits inflammatory responses through direct phosphorylation of the adaptor TRAF6.. Nat Immunol.

[62] Geng J, Sun X, Wang P, Zhang S, Wang X, Wu H, Hong L, Xie C, Li X, Zhao H, Liu Q, Jiang M, Chen Q, Zhang J, Li Y, Song S, Wang HR, Zhou R, Johnson RL, Chien KY, Lin SC, Han J, Avruch J, Chen L, Zhou D (2015). Kinases Mst1 and Mst2 positively regulate phagocytic induction of reactive oxygen species and bactericidal activity.. Nat Immunol.

[63] Li W, Xiao J, Zhou X, Xu M, Hu C, Xu X, Lu Y, Liu C, Xue S, Nie L, Zhang H, Li Z, Zhang Y, Ji F, Hui L, Tao W, Wei B, Wang H (2015). STK4 regulates TLR pathways and protects against chronic inflammation-related hepatocellular carcinoma.. J Clin Invest.

[64] Ea CK, Sun L, Inoue J, Chen ZJ (2004). TIFA activates IkappaB kinase (IKK) by promoting oligomerization and ubiquitination of TRAF6.. Proc Natl Acad Sci U S A.

[65] Wang C, Deng L, Hong M, Akkaraju GR, Inoue J, Chen ZJ (2001). TAK1 is a ubiquitin-dependent kinase of MKK and IKK.. Nature.

[66] Kaufmann SH, Dorhoi A (2016). Molecular Determinants in Phagocyte-Bacteria Interactions.. Immunity.

[67] Gordon S (2016). Phagocytosis: An Immunobiologic Process.. Immunity.

[68] Underhill DM, Goodridge HS (2012). Information processing during phagocytosis.. Nat Rev Immunol.

[69] West AP, Brodsky IE, Rahner C, Woo DK, Erdjument-Bromage H, Tempst P, Walsh MC, Choi Y, Shadel GS, Ghosh S (2011). TLR signalling augments macrophage bactericidal activity through mitochondrial ROS.. Nature.

[70] Mehta D, Rahman A, Malik AB (2001). Protein kinase C-alpha signals rho-guanine nucleotide dissociation inhibitor phosphorylation and rho activation and regulates the endothelial cell barrier function.. J Biol Chem.

[71] Vogel RO, Janssen RJ, van den Brand MA, Dieteren CE, Verkaart S, Koopman WJ, Willems PH, Pluk W, van den Heuvel LP, Smeitink JA, Nijtmans LG (2007). Cytosolic signaling protein Ecsit also localizes to mitochondria where it interacts with chaperone NDUFAF1 and functions in complex I assembly.. Genes Dev.

[72] Liu B, Zheng Y, Yin F, Yu J, Silverman N, Pan D (2016). Toll Receptor-Mediated Hippo Signaling Controls Innate Immunity in Drosophila.. Cell.

[73] Gay NJ, Symmons MF, Gangloff M, Bryant CE (2014). Assembly and localization of Toll-like receptor signalling complexes.. Nat Rev Immunol.

[74] An H, Hou J, Zhou J, Zhao W, Xu H, Zheng Y, Yu Y, Liu S, Cao X (2008). Phosphatase SHP-1 promotes TLR- and RIG-I-activated production of type I interferon by inhibiting the kinase IRAK1.. Nat Immunol.

[75] Yamin TT, Miller DK (1997). The interleukin-1 receptor-associated kinase is degraded by proteasomes following its phosphorylation.. J Biol Chem.

[76] Rakoff-Nahoum S, Medzhitov R (2009). Toll-like receptors and cancer.. Nat Rev Cancer.

[77] Pradere JP, Dapito DH, Schwabe RF (2014). The Yin and Yang of Toll-like receptors in cancer.. Oncogene.

[78] Bald T, Quast T, Landsberg J, Rogava M, Glodde N, Lopez-Ramos D, Kohlmeyer J, Riesenberg S, van den Boorn-Konijnenberg D, Homig-Holzel C, Reuten R, Schadow B, Weighardt H, Wenzel D, Helfrich I, Schadendorf D, Bloch W, Bianchi ME, Lugassy C, Barnhill RL, Koch M, Fleischmann BK, Forster I, Kastenmuller W, Kolanus W, Holzel M, Gaffal E, Tuting T (2014). Ultraviolet-radiation-induced inflammation promotes angiotropism and metastasis in melanoma.. Nature.

[79] Dapito DH, Mencin A, Gwak GY, Pradere JP, Jang MK, Mederacke I, Caviglia JM, Khiabanian H, Adeyemi A, Bataller R, Lefkowitch JH, Bower M, Friedman R, Sartor RB, Rabadan R, Schwabe RF (2012). Promotion of hepatocellular carcinoma by the intestinal microbiota and TLR4.. Cancer Cell.

[80] Tamura T, Yanai H, Savitsky D, Taniguchi T (2008). The IRF family transcription factors in immunity and oncogenesis.. Annu Rev Immunol.

[81] Ikushima H, Negishi H, Taniguchi T (2013). The IRF family transcription factors at the interface of innate and adaptive immune responses.. Cold Spring Harb Symp Quant Biol.

[82] Meng F, Zhou R, Wu S, Zhang Q, Jin Q, Zhou Y, Plouffe SW, Liu S, Song H, Xia Z, Zhao B, Ye S, Feng XH, Guan KL, Zou J, Xu P (2016). Mst1 shuts off cytosolic antiviral defense through IRF3 phosphorylation.. Genes Dev.

[83] Zhang Q, Meng F, Chen S, Plouffe SW, Wu S, Liu S, Li X, Zhou R, Wang J, Zhao B, Liu J, Qin J, Zou J, Feng XH, Guan KL, Xu P (2017). Hippo signalling governs cytosolic nucleic acid sensing through YAP/TAZ-mediated TBK1 blockade.. Nat Cell Biol.

[84] Wang S, Xie F, Chu F, Zhang Z, Yang B, Dai T, Gao L, Wang L, Ling L, Jia J, van Dam H, Jin J, Zhang L, Zhou F (2017). YAP antagonizes innate antiviral immunity and is targeted for lysosomal degradation through IKKvarepsilon-mediated phosphorylation.. Nat Immunol.

[85] Liu Y, Lu J, Zhang Z, Zhu L, Dong S, Guo G, Li R, Nan Y, Yu K, Zhong Y, Huang Q (2017). Amlexanox, a selective inhibitor of IKBKE, generates anti-tumoral effects by disrupting the Hippo pathway in human glioblastoma cell lines.. Cell Death Dis.

[86] Jiao S, Guan J, Chen M, Wang W, Li C, Wang Y, Cheng >, Zhou Z (2017). Targeting IRF3 as a YAP agonist therapy against gastric cancer.. J Exp Med.

[87] Meng Z, Moroishi T, Guan KL (2016). Mechanisms of Hippo pathway regulation.. Genes Dev.

[88] Thompson BJ, Sahai E (2015). MST kinases in development and disease.. J Cell Biol.

[89] Zou Q, Jin J, Xiao Y, Hu H, Zhou X, Jie Z, Xie X, Li JY, Cheng X, Sun SC (2015). T cell development involves TRAF3IP3-mediated ERK signaling in the Golgi.. J Exp Med.

[90] Hoebeke I, De Smedt M, Stolz F, Pike-Overzet K, Staal FJ, Plum J, Leclercq G (2007). T-, B- and NK-lymphoid, but not myeloid cells arise from human CD34(+)CD38(-)CD7(+) common lymphoid progenitors expressing lymphoid-specific genes.. Leukemia.

[91] Huang J, Liu T, Xu LG, Chen D, Zhai Z, Shu HB (2005). SIKE is an IKK epsilon/TBK1-associated suppressor of TLR3- and virus-triggered IRF-3 activation pathways.. EMBO J.

[92] Abdollahpour H, Appaswamy G, Kotlarz D, Diestelhorst J, Beier R, Schaffer AA, Gertz EM, Schambach A, Kreipe HH, Pfeifer D, Engelhardt KR, Rezaei N, Grimbacher B, Lohrmann S, Sherkat R, Klein C (2012). The phenotype of human STK4 deficiency.. Blood.

[93] Nehme NT, Pachlopnik Schmid J, Debeurme F, Andre-Schmutz I, Lim A, Nitschke P, Rieux-Laucat F, Lutz P, Picard C, Mahlaoui N, Fischer A, de Saint Basile G (2012). MST1 mutations in autosomal recessive primary immunodeficiency characterized by defective naive T-cell survival.. Blood.

[94] Fukuhara T, Tomiyama T, Yasuda K, Ueda Y, Ozaki Y, Son Y, Nomura S, Uchida K, Okazaki K, Kinashi T (2015). Hypermethylation of MST1 in IgG4-related autoimmune pancreatitis and rheumatoid arthritis.. Biochem Biophys Res Commun.

